# A Mixture of Persistent Organic Pollutants and Perfluorooctanesulfonic Acid Induces Similar Behavioural Responses, but Different Gene Expression Profiles in Zebrafish Larvae

**DOI:** 10.3390/ijms18020291

**Published:** 2017-01-29

**Authors:** Abdolrahman Khezri, Thomas W. K. Fraser, Rasoul Nourizadeh-Lillabadi, Jorke H. Kamstra, Vidar Berg, Karin E. Zimmer, Erik Ropstad

**Affiliations:** 1 Department of Basic Sciences and Aquatic Medicine, Faculty of Veterinary Medicine, Norwegian University of Life Sciences, P.O. Box 8146 Dep., 0033 Oslo, Norway; rasoul.nourizadeh-lillabadi@nmbu.no (R.N.-L.); jorke.kamstra@nmbu.no (J.H.K.); karin.zimmer@nmbu.no (K.E.Z.); 2 Department of Production Animal Clinical Sciences, Faculty of Veterinary Medicine, Norwegian University of Life Sciences, P.O. Box 8146 Dep., 0033 Oslo, Norway; thomas.fraser@nmbu.no (T.W.K.F.); erik.ropstad@nmbu.no (E.R.); 3 Department of Food Safety and Infection Biology, Faculty of Veterinary Medicine, Norwegian University of Life Sciences, P.O. Box 8146 Dep., 0033 Oslo, Norway; vidar.berg@nmbu.no

**Keywords:** persistent organic pollutants, PFOS, zebrafish larvae, behavioural, neurotoxicity

## Abstract

Persistent organic pollutants (POPs) are widespread in the environment and some may be neurotoxic. As we are exposed to complex mixtures of POPs, we aimed to investigate how a POP mixture based on Scandinavian human blood data affects behaviour and neurodevelopment during early life in zebrafish. Embryos/larvae were exposed to a series of sub-lethal doses and behaviour was examined at 96 h post fertilization (hpf). In order to determine the sensitivity window to the POP mixture, exposure models of 6 to 48 and 48 to 96 hpf were used. The expression of genes related to neurological development was also assessed. Results indicate that the POP mixture increases the swimming speed of larval zebrafish following exposure between 48 to 96 hpf. This behavioural effect was associated with the perfluorinated compounds, and more specifically with perfluorooctanesulfonic acid (PFOS). The expression of genes related to the stress response, GABAergic, dopaminergic, histaminergic, serotoninergic, cholinergic systems and neuronal maintenance, were altered. However, there was little overlap in those genes that were significantly altered by the POP mixture and PFOS. Our findings show that the POP mixture and PFOS can have a similar effect on behaviour, yet alter the expression of genes relevant to neurological development differently.

## 1. Introduction

Persistent organic pollutants (POPs) refers to groups of toxic environmental chemicals with a carbon-based structure, resistant to environmental degradation and widely distributed via soil, water and air [1]. Because of their lipophilic nature, POPs tend to bioaccumulate in top predators and humans [2]. Among different classes of POPs, chlorinated, brominated and perfluorinated compounds are the most persistent compound classes, widely detected in human adipose tissue, breast milk and serum samples from all over the world [3,4,5,6,7]. 

POPs are endocrine disruptors and have been shown to have a wide range of effects including impaired reproduction, carcinogenicity, and thyroid disruption, and can promote cardiovascular disease and induce hepatic lesions [8,9]. Of particular concern is the lipophilic property of POPs that makes them capable of passing through biological barriers such as the placenta [10]. Indeed, several POPs are known to be neurotoxic [11,12,13] and have been associated with neurological diseases in children [14]. The complicated processes taking place during development make the brain and neural tissue sensitive to a variety of environmental contaminants [15,16]. Previous studies have demonstrated the ability of POPs such as perfluorooctanesulfonic acid (PFOS) to pass through the blood−brain barrier [17], causing neurotoxicity and behavioural alterations in mice, rats, and zebrafish [18,19,20,21,22,23]. As for the potential mechanisms, work in zebrafish has demonstrated that POPs such as PFOS can promote cell death in the brain following early life exposure which is then associated with altered behaviour [20]. Moreover, exposure can induce reactive oxidant species (ROS) [24] and estrogenic biomarkers [25], as well as influence the expression of genes related to metabolism and organogenesis [26]. Behavioural responses may also be related to dopaminergic deficits [27].

A large and growing body of literature has been published on the effectiveness of zebrafish as a model organism. These studies all indicate that zebrafish, due to their small size, high offspring rate, rapid development, short generation period, low cost, and transparent embryos, make a successful model organism for high-throughput screening studies [28,29,30]. In addition, recent work has highlighted the use of behaviour as a sensitive tool for assessing the sub-lethal effects of environmental pollutants [31,32,33] on both general toxicity [34] as well as neurotoxicity [31,32,33]. Furthermore, zebrafish have proven to be a useful model system for developmental neurotoxicity and investigating mechanistic pathways. For instance, previous studies have shown how the expression of central nervous system (CNS)-related genes in zebrafish can be impaired following exposure to different compounds [35,36,37,38]. 

The majority of toxicological studies have focused on the effects of single compounds only, whereas in reality we are exposed to complex mixtures of pollutants [39]. Indeed, environmentally relevant mixtures of POPs can induce biomarkers of estrogenic activity and induce cytochrome P4501A [40], impair reproductive function [40] and lead to behavioural aberrations [41,42]. However, less is known about which chemicals within these mixtures are influencing specific endpoints or how such mixtures interact on toxicological endpoints. This is a significant concern as several studies have demonstrated the potential of different compounds to have additive effects. For instance, it has been shown that a combination of 17α-ethinyl estradiol (EE2) and dibutyl phthalate (DBP) had greater effects on gonad, liver and gill development in zebrafish compared to EE2 and DBP exposures alone [43]. Similarly, co-exposing zebrafish larvae to PFOS and nano-ZnO led to more serious thyroid-disrupting effects than exposure to PFOS alone [44]. With this in mind, we recently developed a POP mixture based on Scandinavian blood data. Initial studies have shown that our POP mixture induces ROS production in a human hepatocarcinoma cell line [45]. Furthermore, individual compounds within the POP mixture and not the total POP mixture altered the transcriptional activity of the glucocorticoid receptor in the glucocorticoid receptor redistribution assay [46]. 

Animals and humans are exposed to POPs in a mixture scenario. Therefore, investigating the effects of environmentally relevant POP mixtures is more realistic than the effects of a single POP. Moreover, the research to date has tended to focus on observed behavioural responses following exposure to specific compounds or a group of them, rather than investigating the mechanistic pathways involved in the behavioural response. Therefore, the aim of this study was to investigate the possible neurobehavioural effects of an environmentally relevant POP mixture and sub-mixtures, derived from Scandinavian human blood data, on zebrafish larvae. The secondary aim was to investigate the impact of the POP mixture on the expression of genes relevant to brain development and behaviour during the early life stage of zebrafish.

## 2. Results

### 2.1. Total Persistent Organic Pollutant (POP) Mixture Increased Swimming Speed 

**Figure 1:**
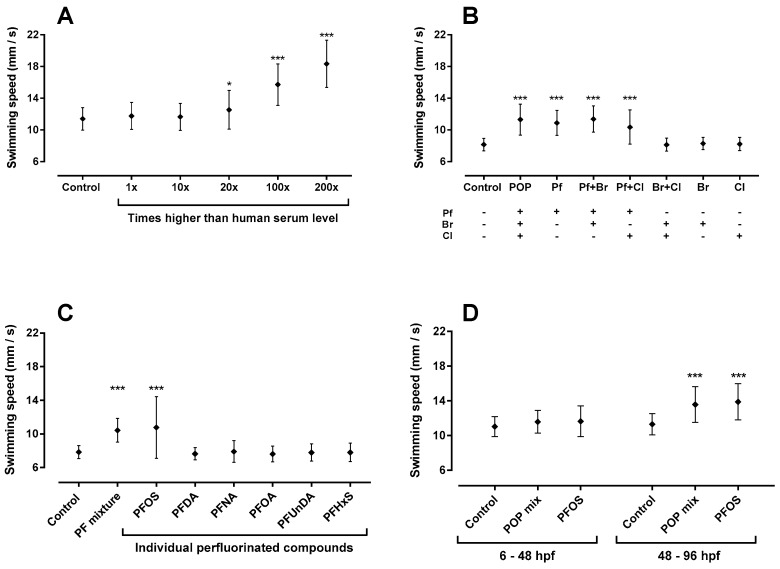
Swimming speed in zebrafish larvae exposed to a mixture of environmental pollutants, sub-mixtures and individual perfluorinated compounds. (**A**) swimming speed in zebrafish larvae upon exposure to five different concentrations of total persistent organic pollutant (POP) mixture; (**B**) swimming speed in zebrafish larvae upon exposure to sub-mixtures at the concentration equal to 100× higher than that found in human serum; (**C**) swimming speed after exposing the zebrafish to individual perfluorinated compounds (100× human serum level) compared to PF mixture; (**D**) PFOS and POPs sensitivity test (100× human serum level). (Pf) Perfluorinated mixture; (Br) Brominated mixture; (Cl) Chlorinated mixture; (Pf + Br) binary mixture of perfluorinated and brominated compounds; (Pf + Cl) binary mixture of perfluorinated and chlorinated compounds; (Br + Cl) binary mixture of brominated and chlorinated compounds. (+) contained; (−) not contained. Data are means ± SD. An asterisk identifies values that are significantly different from the solvent (0.05% DMSO) control (LME, * = *p* < 0.017, ** = *p* < 0.0017, *** = *p* < 0.0001).

The first part of the experiment was to screen the mixture for behavioural effects. We looked at three endpoints: the total distance moved, the total time spent active and the average swimming speed (Appendix A). From these, the average swimming speed was identified as the most robust behavioural response and used for further study. The total POP mixture at an equal concentration to that found in human plasma had no effect on larval swimming speed, while doses 20×, 100× and 200× higher than the human serum level resulted in significant increases in the average swimming speed (10%, 38% and 61% increase, respectively) compared with controls (Figure 1A). Based on the clear response at 100× higher than human serum level, and in order to minimize any possible general toxicity, this concentration was selected for further investigation.

### 2.2. Sub-Mixtures Containing Perfluorinated Compounds Increased Swimming Speed

The total POP mixture consisted of three main sub-mixtures, perfluorinated, brominated and chlorinated compounds. Therefore, the next step was to identify which groups contributed to the observed behavioural response following exposure to the total mixture. It can be seen from the data in Figure 1B that neither the brominated or chlorinated compounds alone or in combination had any effect on swimming speed. However, exposure to mixtures containing perfluorinated compounds, at a concentration equal to 100× higher than human serum level, significantly increased swimming speed similar to what was observed following exposure to the total POP mixture. 

### 2.3. Perfluorooctanesulfonic Acid (PFOS) Increased Swimming Speed 

In order to identify the role of individual perfluorinated compounds in increasing the swimming speed, zebrafish embryos were exposed to the six different chemicals that made up the perfluorinated mixture. We found only PFOS significantly increased swimming speed in zebrafish larvae, similar to both the perfluorinated and total POP mixtures (Figure 1C). 

### 2.4. PFOS Tissue Uptake in Larvae

We found the increase in swimming speed observed after exposure to the total POP mixture was mimicked by PFOS exposure. Based on this finding, we evaluated PFOS accumulation in 96 hpf zebrafish larvae after exposure to the total POP mixture at a concentration equal to 100× the human serum level. Our results showed that after 96 h exposure, 22% of the nominal PFOS concentration was detected. Of this, 49% accumulated in the larvae, 49% remained in the exposure medium and 2% was stuck to the wells. 

### 2.5. 48–96 hpf as Developmental Window of Sensitivity 

Based on our observed results, PFOS was the only compound that could explain the behavioural response in zebrafish larvae exposed to the total POP mixture. Next, we tested which phase of zebrafish neurodevelopmental is the most sensitive to PFOS and POP exposure. We observed that exposure from 48 to 96 hpf significantly increased swimming speed, whereas exposure from 6 to 48 hpf had no effect on swimming speed (Figure 1D). In addition, we observed that the insensitivity between 6 to 48 hpf was not related to the presence of the chorion as exposure between 24 to 48 hpf in dechorionated embryos did not increase swimming speed compared to the control (Appendix B). 

### 2.6. POP Mixture and PFOS Altered Gene Expression Differently

**Figure 2:**
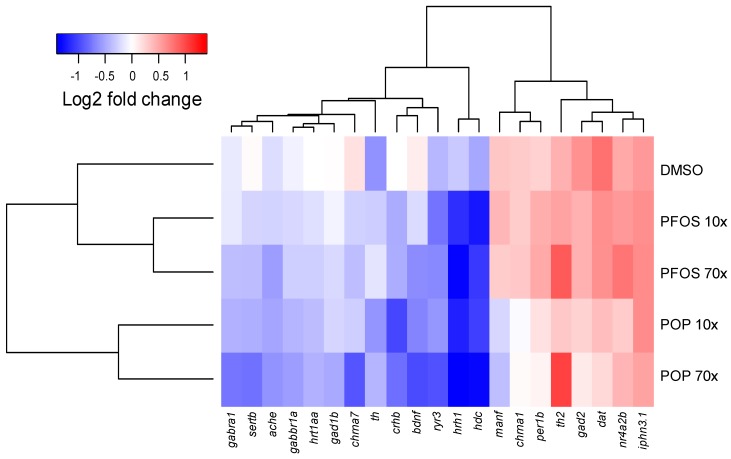
Euclidean distance and ward clustering on log2 normalized expression values. The heat map shows the differences in expression of 21 genes related to neurodevelopmental processes between the solvent control (0.05% DMSO) and exposed samples in 96 hpf zebrafish. Cluster analysis was performed on log2 expression values of five biological replicates.

We investigated the expression of a battery of genes involved in neurodevelopment and behaviour after exposure to the POP mixture and PFOS between 48 to 96 hpf. POP and PFOS exposure led to different gene expression profiles. Cluster analyses revealed that both PFOS 10× and PFOS 70× clustered together as did POP 10× and POP 70×. In addition, the distance between POP-exposed groups and control was greater than the distance between PFOS-exposed groups and the control (Figure 2). 

**Figure 3:**
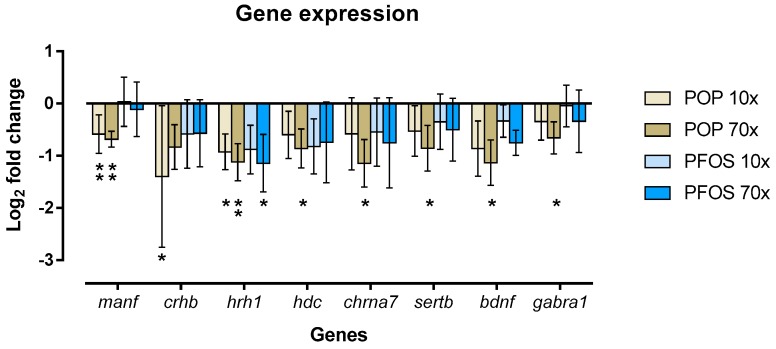
Transcription levels in genes relevant to behaviour following POP mixture and PFOS exposure. The line at zero indicates the gene expression in control groups (DMSO 0.05%). Data are presented as mean ± SD relative to control. An asterisk identifies genes expression levels that were significantly different from the solvent control (one-way ANOVA test, * = *p* < 0.05, ** = *p* < 0.005).

Although differences in gene expression profiles were detected via cluster analysis, the expression of the majority of genes remained unchanged with only eight genes including *manf*, *crhb*, *hrh1*, *hdc*, *chrna7*, *sertb*, *bdnf* and *gabra1* being significantly affected. The POP exposure significantly affected the greatest number of genes, whereas PFOS exposure only affected one gene, *hrh1*. The genes *manf* and *hrh1* were significantly downregulated in both the POP 10× and 70×-exposed larvae. Transcription levels of *hdc*, *chrna7*, *sertb*, *bdnf* and *gabra1* were significantly decreased only in the POP 70× group, whereas *crhb* was significantly affected only in the POP 10× group. Finally, *hrh1* was the only gene that was significantly downregulated by both POP 70× and PFOS 70× exposures (Figure 3). 

## 3. Discussion

Our aim was to determine whether a human POP mixture based on human blood levels from the Scandinavian population could induce behavioural effects following developmental exposure, using zebrafish as a model vertebrate system. Our results indicated that the total POP mixture significantly affected the swimming behaviour in zebrafish larvae starting at a concentration 20× higher than that found in human serum. Further investigations revealed that PFOS alone could mimic the behavioural response observed following exposure to the POP mixture. However, although the results from gene expression analysis revealed that both the POP mixture and PFOS altered the regulation of CNS-related genes, there was limited overlap in those genes significantly affected. Our work highlights the potential developmental neurotoxicity of a POP mixture relevant to humans. To date, very little attention has been paid to the potency of mixtures of environmental pollutants on the induction of neurobehavioural toxicity. Previous work would suggest the results of single compounds are not fully translatable to mixture scenarios, mainly because of unknown interactions between different chemicals in complex mixtures [47]. We found the POP mixture increased the swimming speed in zebrafish larvae in a dose-dependent manner. This behavioural effect was associated with the perfluorinated compounds within the mixture, more specifically with PFOS. This result could be explained by the fact that PFOS was the compound with the highest concentration in the total and perfluorinated mixtures (5.46 µM in 100× mixtures), compared with PFOA (1 µM in 100× mixture) which was the second most concentrated compound. Of note, PFOS alone increased swimming speed to a similar extent as the total POP mixture, which suggests PFOS toxicity was not influenced by other compounds in the POP mixture. Previous studies have shown a hyperactive behaviour upon PFOS exposure in both zebrafish and rodents. For instance, zebrafish larvae exposed to 1.85 µM PFOS developed spontaneous activity and persistent hyperactivity [27]. Another study reported that PFOS in a wide range of concentrations (0.5 to 8 µM) increases the swimming speed in both 5 and 6 dpf zebrafish larvae [20]. It has also been shown that chronic prenatal exposure to PFOS (0.5 µM) for 120 days in zebrafish is able to increase the swimming speed in both parents and F1 larvae [18]. Similarly, mice exposed to 3 mg/kg/day PFOS displayed spontaneous activity [27] whereas other rodent studies have found that PFOS decreases locomotor activity [48,49]. 

Regarding the increase in swimming speed, we found that 48–96 hpf is the sensitive window for the total POP mixture and PFOS exposure, as exposures before 48 hpf had no effect on swimming speed. These results match those observed in earlier studies. For example, it has been shown that zebrafish larvae exposed to PFOS from 49 to 73 hpf had higher swimming speeds compared with groups exposed before 49 hpf and after 73 hpf [20]. Moreover, it has been reported that 16 µM PFOS exposure between 48 to 96 hpf in zebrafish larvae resulted in noticeable deformities (uninflated swim bladder, less developed gut, and curved spine), whereas larvae developmentally exposed to PFOS from 8 to 48 hpf did not develop any distinct deformities, even after exposure to 32 µM [26]. Another study reported that PFOS exposure before 48 hpf had no effect on the development of the swim bladder, while exposure after 48 hpf resulted in swim bladder deformities in 50% of the zebrafish larvae [50]. Different hypotheses have been suggested regarding the sensitivity of zebrafish larvae to PFOS exposure. For instance, this window of sensitivity might be related to the development of estrogenic receptors, which begin to be expressed after 48 hpf in zebrafish larvae, and could mediate PFOS toxicity [26]. However, although PFOS exposure does produce estrogenic effects in zebrafish [25], we have previously found exposure to 10 nM of the xenoestrogen 17α-ethinylestradiol (EE2) has no effect on behaviour at 96 hpf even though we detected an elevation in the expression of estrogenic response genes [51]. Furthermore, it seems that PFOS toxicity is not related to the presence of the chorion. Previous work has demonstrated that PFOS accumulates in 6 hpf-exposed embryos two hours after exposure, but absorption and accumulation of PFOS is accelerated in larvae after 48 hpf [20]. This increase in absorption at later life stages may explain why larvae were more sensitive to PFOS exposure at the later life stage.

We evaluated gene transcription after POP and PFOS exposure during the 48–96 hpf window. As previously reviewed [29,52,53], the different regions of the zebrafish brain are almost developed by 48 hpf and between 48–96 hpf the developmental processes for different neurotransmitter-expressing neurons is accelerating. Therefore, we hypothesized that those CNS processes that start to develop after 48 hpf in zebrafish larvae could mediate the POP and PFOS behavioural toxicity. Although both POP 70× and PFOS 70× exposure significantly increased the swimming speed, we found only one mutually affected gene (*hrh1*) between these exposure groups, whereas other genes involved in dopaminergic (*manf*), histaminergic (*hdc*), serotonergic (*sertb*), cholinergic (*chrna7*), GABAergic (*gabra1*), stress (*crhb*) and neural maintenance (*bdnf*) signalling were exclusively affected in POP-exposed groups. This could be explained by presence of brominated, chlorinated and perfluorinated compounds within the mixture. Similarly, mixtures of polycyclic aromatic hydrocarbons (PAHs) had limited overlap on gene expression compared to individual compounds in rat liver [54]. The cluster analysis confirmed that the POP exposure altered the gene expression profile in a different manner compared with PFOS. This would suggest that the genes assessed here were either not involved in the observed behavioural responses or that PFOS has a different molecular pathway leading to the observed behavioural effects. 

Previous studies have implemented the dopaminergic and serotonergic systems in the neurotoxicity of PFOS, but we found no clear evidence that these systems explained the increase in swimming speed in the current study. For example, PFOS increased the level of serotonin in different regions of the rat brain [55] and impaired the dopaminergic system in both mice and zebrafish [22,27]. Moreover, it has been shown that PFOS exposure upregulated *crhb*, which is a marker of the stress response [56]. However, the dopaminergic genes *sertb*, and *crhb* were not significantly affected by PFOS in this study. Additionally, although gene expression was more influenced by exposure to 70× compared to 10× of the POP mixture, including genes involved in inhibitory signaling pathways (*sertb*, *gabra1*) [57,58], it is unclear which systems may be behind the behavioural effects observed in the current study.

One of the main objectives of toxicity testing is to determine the lowest effect concentrations. Gene analyses data revealed that *manf*, *crhb* and *hrh1* genes were significantly downregulated upon POP exposure, even at a concentration only 10× higher than human serum level. *manf* is a dopaminergic neurotrophic factor that protects dopaminergic neurons from neurotoxic damage [59] and plays a supportive role in cell viability [60]. In addition to the stress response, *crhb* also plays an important role in thyroid-stimulating hormone (TSH) secretion [61]. There was also significant downregulation of *crhb* in this study, thereby suggesting a possible disruptive effect of the POP mixture on the hypothalamic-pituitary-interrenal (*HPI*)/hypothalamic-pituitary-adrenal (*HPA*), and hypothalamic-pituitary-thyroid (*HPT*) axis. *Hrh1* is a histamine receptor expressed widely in the CNS, and also regulates the immune response [62]. Therefore, although no behavioural effect was observed following the 10× exposure, changes in gene expression were observed at concentrations close to the human scenario. Further research is needed to explore the biological significance of these changes in gene expression and which compounds from the POP mixture are responsible for these changes. 

Based on our results, exposure to a mixture of brominated and/or chlorinated compounds had no effect on swimming speed. Similarly, it has been reported that brominated compounds including BDE 47, 99, 100, 153, 154, 209 and HBCD, at concentrations within the range used in current study, had no significant effect on locomotor behaviour in 5 dpf zebrafish larvae [63]. Although some brominated and chlorinated compounds are known to influence larval zebrafish behaviour in contrast to our results, these compounds are either not in our mix or the effects were found at concentrations higher than those used in the current study [42,64]. 

We used larval zebrafish to assess a human-based POP mixture for behavioural effects. Differences in larval locomotor behaviour using the light/dark assay are generally associated with the level of anxiety [65], suggesting our mixture could lead to alterations in anxiety within humans. The concentrations tested were of relevance to humans, as we found effects levels only marginally higher (i.e., 10×) than those found in human blood serum. Here it is noted that the human-based POP mixture was based on the mean values within Scandinavians. Therefore, some individuals will have higher values than the mean, and the levels of environmental pollutants within humans varies between different countries and tends to be lower in more developed countries [66]. Furthermore, we could only recover 22% of the nominal value for PFOS at 96 hpf. It is unclear where the remaining 78% went, but the values attained per embryo (63 ng following exposure to 5.5 µM PFOS) were very similar to those values obtained by [20] following exposure between 0 and 5 days post fertilization (66 ng/embryo following exposure to 8 µM PFOS).

## 4. Materials and Methods 

### 4.1. Mixtures and Chemicals 

The POP mixtures were designed and made by the Norwegian University of Life Sciences, Oslo, and described in [67]. Relevant compounds and their levels in human plasma of a Scandinavian population were identified, and seven different mixtures were prepared and used in the current study, including; (1) total POP mixture containing perfluorinated, brominated and chlorinated compounds; (2) perfluorinated mixture (Pf); (3) brominated mixture (Br); (4) chlorinated mixture (Cl); (5) perfluorinated and brominated mixture (Pf + Br); (6) perfluorinated and chlorinated mixture (Pf + Cl); and (7) brominated and chlorinated mixture (Br + Cl). The compounds were mixed in concentration ratios relevant to the human serum level. The intention was that the dose of each mixture reflected the human plasma level of corresponding chemicals within that mixture. The chemicals included in the mixtures and their respective concentrations are shown in Table S1. All polybrominated diphenyl ethers (PBDEs), polychlorinated biphenyls (PCBs) and other organochlorines were originally purchased from Chiron As (Trondheim, Norway). Hexabromocyclododecane (HBCD) and all perfluorinated compounds (PFCs) were obtained from Sigma-Aldrich (St. Louis, MO, USA), except PFHxS which was from Santa Cruz (Dallas, TX, USA). All stock solutions were formed in pure DMSO (Sigma-Aldrich).

### 4.2. Zebrafish Maintenance and Breeding

Adult AB strain zebrafish (*Danio rerio*) were housed with a 14:10 h light:dark cycle period in a carbon-filtered flow-through system. System water was kept at 28 ± 1 °C and prepared by adding 15.5 g of Instant Ocean^®^ salt, 5.3 g of sodium bicarbonate and 1.5 g of calcium chloride per 100 L of tap water to attain a pH of 7.5–7.6 and conductivity of 500 µS/cm. Fish were fed daily, twice with Artemia and once with formulated feed (SDS 400, Essex, UK) and kept at a density equal to seven fish/L. For egg production, male and female adult zebrafish were held in breeding tanks equipped with a barrier and spawning net. The barriers were removed shortly after the onset of light in the morning and the fish paired for 30 min. Eggs collected from the breeding tanks were rinsed and kept in autoclaved system water at 28 °C until exposure.

### 4.3. Exposure Scenario 

The study was performed at the Section for Experimental Biomedicine at the Norwegian University of Life Sciences in Oslo, Norway. The unit is licensed by the Norwegian Food Inspection Authority (NFIA) and accredited by the Association for Assessment and Accreditation of Laboratory Animal Care (2014/225976). The study (2013/39783-2) was approved on 20/08/2013 by the unit’s animal ethics committee (Institutional Animal Care and Use Committee/IACUC) and NFIA. 

Fertilized and healthy embryos at approximately 6 hpf were selected using a stereo microscope. Equal numbers of embryos for each treatment were distributed in a checker-box pattern across 96-well plates (Thermo Fisher Scientific, Roskilde, Denmark) (one embryo/well) and exposed statically in 200 µL of media. The final concentration of DMSO in all test concentrations and the solvent control was 0.05%. First, embryos were exposed separately to all seven mixtures over the concentration range 1× to 200× higher than human serum levels (three replicates). These concentrations were considered non-teratogenic based on a maximum mortality/deformity rate of 10% in any one group. These experiments were then followed by exposing the zebrafish embryos to individual chemicals from the PF mixture, including: perfluorooctanoic acid (PFOA), perfluorooctanesulfonic acid potassium salt (PFOS), perfluorodecanoic acid (PFDA), perfluorononanoic acid (PFNA), perfluorohexane sulfonate potassium salt (PFHxS), perfluoroundecanoic acid (PFUnDA) and the perfluorinated mixture itself at a concentration equal to 100× higher than human serum levels (three replicates). After each exposure, plates were placed into sealed transparent plastic bags and kept at 28 °C on a 14:10 h light: dark cycle until 96 hpf, when behavioural tests were undertaken. 

### 4.4. Locomotor Activity 

Behavioural assays were conducted on 96 hpf larvae during a light/dark/light cycle using a Viewpoint Zebrabox (Viewpoint Life Science, Lyon, France). This system consists of a 25-frame per second camera equipped with an infrared filter that is capable of tracking zebrafish movement through its supplied software (Video-Track software, ViewPoint Life Science, France). All tests were performed at 28 °C between 09:00 and 10:30. The test consisted of 10 min of acclimation when the light intensity was set to 100% (these data were excluded from final analyses), followed by a further 10 min of 100% light, 10 min of complete darkness, and a final 10 min of 100% light. Only locomotor activity during the dark period was analysed, as movement during the lighted periods was minimal as expected for this life stage [68]. Zebrafish larvae were distinguished from the background by introducing a 30-pixel threshold difference within the tracking software. In addition, short and large movements were defined as 5 and 8 mm per sec in the protocol, respectively. The total distance moved (mm) and the total time spent active (s) were recorded every 60 s. From this data, the mean swimming speed (mm/s) was calculated. Following locomotor assessment, larvae were evaluated using a microscope for any dead or malformed (spinal/tail aberrations, yolk sac or cardiac edema, aberrations in pigmentation, and loss of equilibrium) individuals to be excluded from behavioural analyses. 

### 4.5. PFOS Tissue Uptake in Larvae

The analyses of embryos, medium and wells was done at the laboratory of Environmental Toxicology at the Norwegian University of Life Sciences. The laboratory is accredited by the Norwegian accreditation for testing PFOS in biological material according to the requirements of the NS-EN ISO/IEC 17025 (TEST 137). The PFOS concentrations in the exposure media and whole-body tissues of zebrafish larvae were measured in embryos exposed from 6 to 96 hpf to the total POP mixture at a concentration equal to 100× higher than in human serum. Six zebrafish larvae from six individual wells were pooled as one sample and the exposure media was taken from the corresponding six wells (200 µL/well). Each well was then rinsed by methanol, which was then collected to measure any chemicals that may have resided on the wall of the well. PFOS was analysed according to [69] and references therein. Both linear and branched PFOS were included in analyses as recommended by [70]. The samples were extracted with methanol and clean up was accomplished using active carbon (EnviCarb, Supelco, Zwijndrecht, The Netherlands). Analysis was performed by the separation of compounds on a high-performance liquid chromatographer (HPLC) with a Discovery C18 column, connected to a C18 pre-column (Supelco, Sigma-Aldrich) and detection with liquid chromatography tandem mass spectrometry (MS-MS) (API 3000, LC/MS/MS System). The details of the analytical quality system have been described in [71]. Briefly, every analytical series included three procedural blanks (solvents), one blind (non-spiked clean), and two spiked clean samples for recoveries. The quality control parameters were within the accepted ranges for the method.

### 4.6. Developmental Sensitivity Test

In order to link the observed behavioural response with the neurodevelopmental stages, we exposed zebrafish embryos at two different time points. Zebrafish embryos were exposed to PFOS and then a total POP mixture at a concentration equal to 100× higher than human serum level from 6 to 48 hpf before being washed three times with autoclaved system water and exposed to 200 µL of the vehicle solution only (DMSO 0.05%) from 48 to 96 hpf. Simultaneously, on the same plate, another group of zebrafish embryos were exposed to 200 µL of vehicle solution during the first 48 h and then the exposure followed between 48–96 hpf by adding PFOS or the total POP mixture at 100× higher than the human serum level. This experiment was repeated in triplicate. 

### 4.7. Gene Transcription Analysis

To determine the mRNA expression induced by the total POP mixture and PFOS, zebrafish larvae were exposed between 48–96 hpf. Concentrations were adjusted to 10× and 70× human serum level as the highest dose at which had no effect on swimming speed (HNSS) or the lowest dose at which there was a significant increase in swimming speed (LISS), respectively (Appendix C). Primers were designed to span exon-exon boundaries using Primer3-based algorithms available at (https://www.ncbi.nlm.nih.gov/tools/primer-blast/) and tested for dimers and efficiency using Vector NTI^®^ advance software version 11 for windows and melting curve, respectively (Table S2). Total RNA was isolated from a pool of 10 embryos from each treatment using Trizol agent (Invitrogen, Carlsbad, CA, USA) and following the manufacturers’ instruction. RNA concentration was measured by nanodrop, cDNA was prepared from 1 μg of DNase-treated total RNA using Superscript III reverse transcriptase (Invitrogen) and random hexamer primers according to product specifications. Quantitative PCR (qPCR) was carried out on a LightCycler^®^ 96 Real-Time PCR system (Roche, Mannheim, Germany) using LightCycler^®^ 480 SYBR Green I Master (Roche). Each cDNA sample was analysed in duplicate and composed of 5 μL mastermix, 2 μL primer mix (5 μM of each of forward and reverse), and 3 μL of each 10× diluted cDNA sample in a total volume of 10 μL. The cycling parameters were 10 min pre-incubation at 95 °C, followed by 45 cycles of amplification at 95 °C for 10 s, 60 °C for 10 s and 72 °C for 15 s, followed by a melting curve from 60 °C to 95 °C. qPCR assay was performed for five biological replicates. After the assessment of candidate reference genes (*hprt*, *rps18*, *ef1α*, *hmbs* and *bactin*) using the online RefFinder analysis available at (http://fulxie.0fees.us/), and based on the Genorm algorithm, *rps18* and *ef1α* were considered the most stable housekeeping genes for all exposure groups. The expression of each target gene transcript was normalized to the housekeeping genes and the fold change was calculated using the ∆∆*C*_t_ method, using the geometric averaging of the two reference genes [72]. 

### 4.8. Statistical Analyses 

Locomotor activity data were transferred to R Studio (RStudio Team 2015, version 0.99.473 for windows, Boston, MA, USA, available at: http://www.rstudio.com/) for behavioural analyses. To test the effect of the total POP mixture, sub-mixtures and individual compounds on locomotor activity, a linear mixed effect model (LME) was employed with distance moved, time spent swimming or swimming speed as the dependent variable, mixture/compound concentration as a categorical independent variable, and test replicate as a random effect. Examination of the residual plots verified that no systematic patterns occurred in the errors (e.g., q-q plots). To assess individual doses to the controls, we used the contrast results provided within R. Due to multiple comparisons of the same data set (i.e., the same individuals were used to assess three behavioural endpoints, distance moved, time active, and swimming speed), the results were Bonferroni corrected to avoid Type I errors. Therefore, significance was assigned at *p* < 0.017 (i.e., 0.05/3). Gene expression data were analysed using one-way ANOVA test followed by Dunnett’s post hoc test and the limit of significance was set at *p* < 0.05. Data were plotted using GraphPad Prism version 7.02 for Windows, (GraphPad Software, San Diego, CA, USA).

## 5. Conclusions 

We aimed to assess the possible neurobehavioural toxicity of an environmentally relevant mixture of persistent organic pollutants (POPs), which was constructed based on Scandinavian human blood data. This study has shown that exposure to a complex mixture consisting of brominated, chlorinated and perfluorinated compounds, significantly affected the swimming speed of zebrafish larvae. The effect was related to the perfluorinated compounds, exclusively with perfluorooctanesulfonic acid (PFOS). These behavioural effects could not be associated with the difference in gene expression. Since behaviour is a complicated phenomenon, further work should investigate whether the POP mixture and PFOS affect additional molecular and physiological processes related to behaviour such as the sensory system or endocrine hormone levels, and investigate the functional role of the genes affected by the POP mixture. 

## Figures and Tables

**Figure B1:**
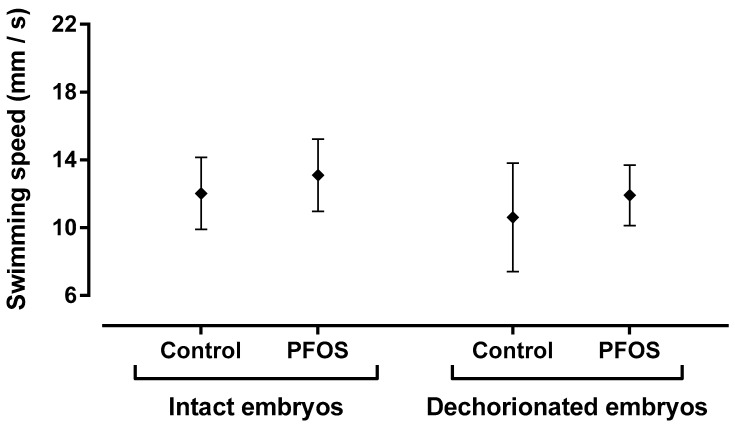
Swimming speed at 96 hpf in zebrafish larvae. Dechorionated and intact zebrafish embryos were exposed to perfluorooctanesulfonic acid (PFOS) (100× human serum level) between 24 to 48 hpf. Data presented as mean ± SD.

**Figure C1:**
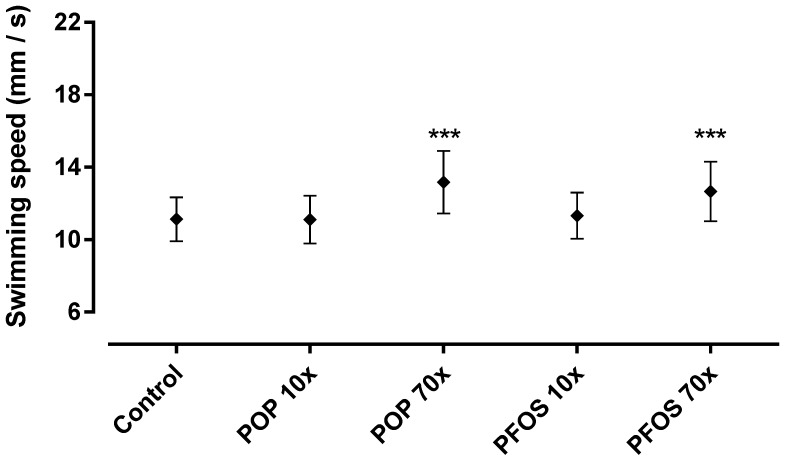
Swimming speed in 96 hpf zebrafish larvae after exposure to POP and PFOS at two different concentrations (10× and 70× higher than human serum level) between 48 to 96 hpf. In order to expose the zebrafish for gene expression analyses, concentrations were adjusted to 10× and 70× human serum level as the highest dose at which there was no significant effect on swimming speed (HNSS) or the lowest dose that consistently and significantly increased the swimming speed (LISS), respectively. Data presented as mean ± SD. An asterisk indicates a significant difference between the exposure group and the solvent control (LME, * = p < 0.017, ** = p < 0.0017, *** = p < 0.0001).

## Supplementary Materials

Supplementary materials can be found at www.mdpi.com/1422-0067/18/2/291/s1.

## Abbreviations

BDE	Brominated diphenyl ethers
BDNF	Brain-derived neurotrophic factor
CHRNA7	Cholinergic receptor nicotinic alpha 7 subunit
CNS	Central nervous system
CRHB	Corticotropin releasing hormone Beta
DMSO	Dimethyl sulfoxide
DPF	Day post fertilization
GABRA1	Gamma-Aminobutyric Acid Type A Receptor Alpha1 Subunit
HDC	Histidine decarboxylase
HPF	Hour post fertilization
HRH1	Histamine Receptor H1
LME	Linear mixed effect
MANF	Mesencephalic astrocyte-derived neurotrophic factor
PBDE	Polybrominated diphenyl ethers
PCB	Polychlorinated biphenyl
PFDA	Perfluorodecanoic acid
PFHxS	Perfluorohexane sulfonate
PFNA	Perfluorononanoic acid
PFOA	Perfluorooctanoic acid
PFOS	Perfluorooctanesulfonic acid
PFUnDA	Perfluoroundecanoic acid
POPS	Persistent organic pollutants
ROS	Reactive oxygen species
SERTB	Serotonin transporter B

## Appendix A

**Table A1:** Distance moved and swimming time in 96 hpf zebrafish larvae exposed to total POP mixture, sub mixtures and individual mixtures.

Exposure Groups	Distance Moved (mm/10 min)						Swimming Time (s/10 min)					
	Control	1×	10×	20×	100×	200×	Control	1×	10×	20×	100×	200×
**Total POPs**	742.1 ± 290.2	782.5 ± 373.4	735.9 ± 303.9	783.2 ± 271.7	722.1 ± 366.2	596.3 ± 400.8 *	66.1 ± 27.1	67.2 ± 31.9	64.1 ± 25.3	64.6 ± 23.8	48.3 ± 26.8 **	34 ± 24.3 ***
**Pf**	730.8 ± 345.3	730.5 ± 371.7	774.4 ± 385.3	722.8 ± 370	743.6 ± 306.7	552.4 ± 367.4 *	61.7 ± 29.8	61 ± 32.3	66.2 ± 34.6	60.2 ± 31.7	47.6 ± 23.9 *	32.6 ± 24.1 ***
**Pf + Br**	784.8 ± 420.4	720.4 ± 326.2	672.8 ± 368.9	834.1 ± 331.1	657.2 ± 330.9	457.2 ± 321 ***	66.9 ± 35.3	63.3 ± 29.3	55.7 ± 31.3	69.2 ± 28.4	42.9 ± 24 ***	25.4 ± 18.5 ***
**Pf + Cl**	667.4 ± 330.8	689.4 ± 305.7	675.9 ± 276.3	669.2 ± 313.3	632.4 ± 369.2	494.5 ± 313.8 *	57.8 ± 27.3	59 ± 27	57.3 ± 24.7	54.9 ± 24.4	42 ± 28.7 *	30.7 ± 23.9 ***
**Br**	622.5 ± 309.6	562.6 ± 238.3	614.9 ± 283.4	622.8 ± 293.6	656.8 ± 238.3	679.6 ± 297.2	60.3 ± 30.1	52.4 ± 23.2	56.9 ± 27.3	56.7 ± 27.2	59.9 ± 23.4	62.6 ± 27.8
**Cl**	770.6 ± 341.4	684.1 ± 310.5	711.9 ± 269.8	738.1 ± 330.8	889.7 ± 374.6	838.8 ± 403.6	68.4 ± 30.1	58 ± 27.2	60.5 ± 24.4	64.1 ± 29.8	77 ± 32.6	71.3 ± 33.7
**Br + Cl**	677.7 ± 338.2	627.4 ± 319.9	641.7 ± 342.3	654.4 ± 329.8	640 ± 327.3	706.4 ± 277.5	62.6 ± 31.9	58.4 ± 31.4	58.1 ± 32.8	58.4 ± 29.4	57.7 ± 30.6	64.2 ± 26.9

The distance moved and swimming time by zebrafish larvae at 96 hpf after exposure to the total persistent organic pollutants (POP) mixture and different sub-mixtures at different concentrations (1 to 200× higher than human serum level). (Pf) perfluorinated mixture; (Pf + Br) co-mixture of perfluorinated and brominated compounds; (Pf + Cl) co-mixture of perfluorinated and chlorinated compounds; (Br) brominated mixture; (Cl) chlorinated mixture; (Br + Cl) co-mixture of brominated and chlorinated compounds. Data presented as means ± SE. Asterisk indicates a significant exposure effect compared to the control (LME, ** = p* < 0.017, *** = p* < 0.0017, **** = p* < 0.0001).
