# Supplementary material for: A Mixture of Persistent Organic Pollutants and Perfluorooctanesulfonic Acid Induces Similar Behavioural Responses, but Different Gene Expression Profiles in Zebrafish Larvae
Source: Int J Mol Sci. 2017 Jan 29;18(2):291. doi: 10.3390/ijms18020291 (PMC5343827; doi:10.3390/ijms18020291)
Supplement: Supplementary file 1 [file ijms-18-00291-s001.pdf]

Supplementary Materials: A Mixture of Persistent Organic Pollutants and Perfluorooctanesulfonic Acid Induce Similar Behavioural Responses, but Different Gene Expression Profiles in Zebrafish Larvae

Abdolrahman Khezri, Thomas W. K. Fraser, Rasoul Nourizadeh-Lillabadi, Jorke H. Kamstra, Vidar Berg, Karin E. Zimmer and Erik Ropstad

Table S1. The composition and concentrations of chemicals in the persistent organic pollutant (POP) mixture. The value reflect the concentrations equal to 1× the mean human plasma level. (Pf) Perfluorinated mixture; (Br) Brominated mixture; (Cl) Chlorinated mixture; (Pf + Br) binary mixture of perfluorinated and brominated compounds; (Pf + Cl) binary mixture of perfluorinated and chlorinated compounds; (Br + Cl) binary mixture of brominated and chlorinated compounds. The table is adapted from [67].

Compounds	Concentration (nM)						
	Total	Pf	Br	Cl	Pf + Br	Pf + Cl	Br + Cl
**Perfluorinated compounds (PFCs)**							
**PFOA**	10.923	10.923			10.923	10.923	
**PFOS**	54.801	54.801			54.801	54.801	
**PFDA**	0.962	0.962			0.962	0.962	
**PFNA**	1.723	1.723			1.723	1.723	
**PFHxS**	7.873	7.873			7.873	7.873	
**PFUnDA**	0.990	0.990			0.990	0.990	
**Polybrominated diphenyl ethers (PBDEs)**							
**BDE-47**	0.018		0.018		0.018		0.018
**BDE-99**	0.007		0.007		0.007		0.007
**BDE-100**	0.003		0.003		0.003		0.003
**BDE-153**	0.001		0.001		0.001		0.001
**BDE-154**	0.003		0.003		0.003		0.003
**BDE-209**	0.011		0.011		0.011		0.011
**HBCD**	0.038		0.038		0.038		0.038
**Chlorinated compounds (CLCs) including**							
**Polychlorinated biphenyls (PCBs)**							
**PCB 28**	0.050			0.050		0.050	0.050
**PCB 52**	0.034			0.034		0.034	0.034
**PCB 101**	0.024			0.024		0.024	0.024
**PCB 118**	0.196			0.196		0.196	0.196
**PCB 138**	0.615			0.615		0.615	0.615
**PCB 153**	1.003			1.003		1.003	1.003
**PCB 180**	0.490			0.490		0.490	0.490
**Other organochlorines**							
**p,p′-DDE**	1.578			1.578		1.578	1.578
**HCB**	0.410			0.410		0.410	0.410
**α-chlordane**	0.026			0.026		0.026	0.026
**Oxy-chlordane**	0.051			0.051		0.051	0.051
**Trans-nonachlor**	0.092			0.092		0.092	0.092
**α-HCH**	0.020			0.020		0.020	0.020
**β-HCH**	0.182			0.182		0.182	0.182
**γ-HCH (Lindane)**	0.020			0.020		0.020	0.020
**Dieldrin**	0.063			0.063		0.063	0.063

Table S2. Primer sequences for qPCR.

Gene	Accession Number	Sequence	PCR Product Size
Bactin	FJ_915059.1	F: CGAGCAGGAGATGGGAAC<br>R: CAACGGAAACGCTCATTGC	101
Hprt	NM_212986.1	F: CAGCGATGAGGAGCAAGGTTATG<br>R: GTCCATGATGAGCCCGTGAGG	102
Ef1α	AM_422110.2	F: TTGAGAAGAAAATCGGTGGTGCTG<br>R: GGAACGGTGTGATTGAGGGAAATTC	90
Rps18	NM_173234.1	F: CATCCCAGAGAAGTTTCAGCACATC<br>R: CGCCTTCCAACACCCTTAATAGC	104
Hmbs	NM_201154.1	F: GTGTGTGGAATTGGACAACAAAGTG<br>R: CGAGGGCTGATGATGAGATATTGC	91
th1	NM_131149.1	F: TGGATCAGGATCACCCAGGA<br>R: GTAGACCTCCCGCCATGTTC	149
th2	NM_001001829.1	F: CGTTCCGGGTTTTCCAGTGT<br>R: CGAGACGAGTCCAATCTGTGAA	152
manf	NM_001076629	F: AGAGTGTGAAGTCTGTGTGGG<br>R: CGCTGTCAAACTTGACGTTGT	77
hdc	NM_001102593.1	F: CTGGGCTCCACTGGTGTG<br>R: CTTGGACGGGTTGAAGACGA	141
hrh1	NM_001042731.1	F: CGACCTCCACATGTTCACCA<br>R: CGTTGCAGAGCGGGTAAATG	77
crhb	NM_001007379.1	F: CAATTACGCACAGATTCTCCTCG<br>R: GAAGTACTCCTCCCCCAAGC	197
bdnf	NM_001308649.1	F: GGACACTTTCGAGCAGGTCA<br>R: CTCCAAAGGCACTTGGTTGC	178
nr4a2b	NM_001002406.1	F: CGTACAGATCCAACCTGCCA<br>R: TATGGTGAGAGCGGCTATGC	194
iphn3.1	XM_005170940.2	F: GAACAGCTCAGCGACTCTCA<br>R: TGTAGGAGGCTTGGGTGTTG	161
per1b	NM_212439.2	F: AACGCTAAAGGTCCGTCTGT<br>R: CTTGTCCCCAACATGGACGA	141
ache	NM_131846.1	F: CTCCAGGAACACTAGGCTGG<br>R: TACACAGCACCATGCGAGTT	73
gabra1	NM_001077326.1	F: AGCCATCCTGATTTTTCGAGGG<br>R: AGCTTTTTCCAGCCAGAGCA	121
chrna1	NM_131445.1	F: CTCGACCGACCTCTGGAAAC<br>R: GCAGGTCGAAGGGAAAGTGA	176
chrna7	NM_201219.2	F: GAGTGGGACCTTGTGGAAGT<br>R: TCCGCATCACCACCGTAAAA	100
gad2	NM_001017708.2	F: ATTGGCTAACCTCCACTGCC<br>R: CGAGCCAGTAGCATGGCATA	184
gad1b	NM_194419.1	F: CTGTGACACCTGTGACTCCGTA<br>R: GTGTGCAACCCCGTACCAC	181
gabbr1a	XM_689405.6	F: AACAACCGGAGCGACATTCT<br>R: CAGATTCCACATTCGCGCTG	189
htr1aa	NM_001123321.1	F: CTACTCAACTTTCGGGGCGT<br>R: CACCGCCAAGCATTTATCCG	145
sertb	NM_001177459.1	F: ACCCTGCCATATGTTGTGCT<br>R: AGCTGCATCTACCCATACGC	135
ryr3	XM_009294773.1	F: GAGGCAACGTTCTTGTGCAG<br>R: CCGTCCTTTCACGCTGATTG	191
dat	NM_131755.1	F: TCAAGTTCCTGCACAAACATCG<br>R: CACAAATTTCCAGCACAGTCTCC	268
